# Supplementary figures and images for: The prognostic model of low-grade glioma based on m6A-associated immune genes and functional study of FBXO4 in the tumor microenvironment
Source: PeerJ. 2025 Mar 21;13:e19194. doi: 10.7717/peerj.19194 (PMC11932111; doi:10.7717/peerj.19194)

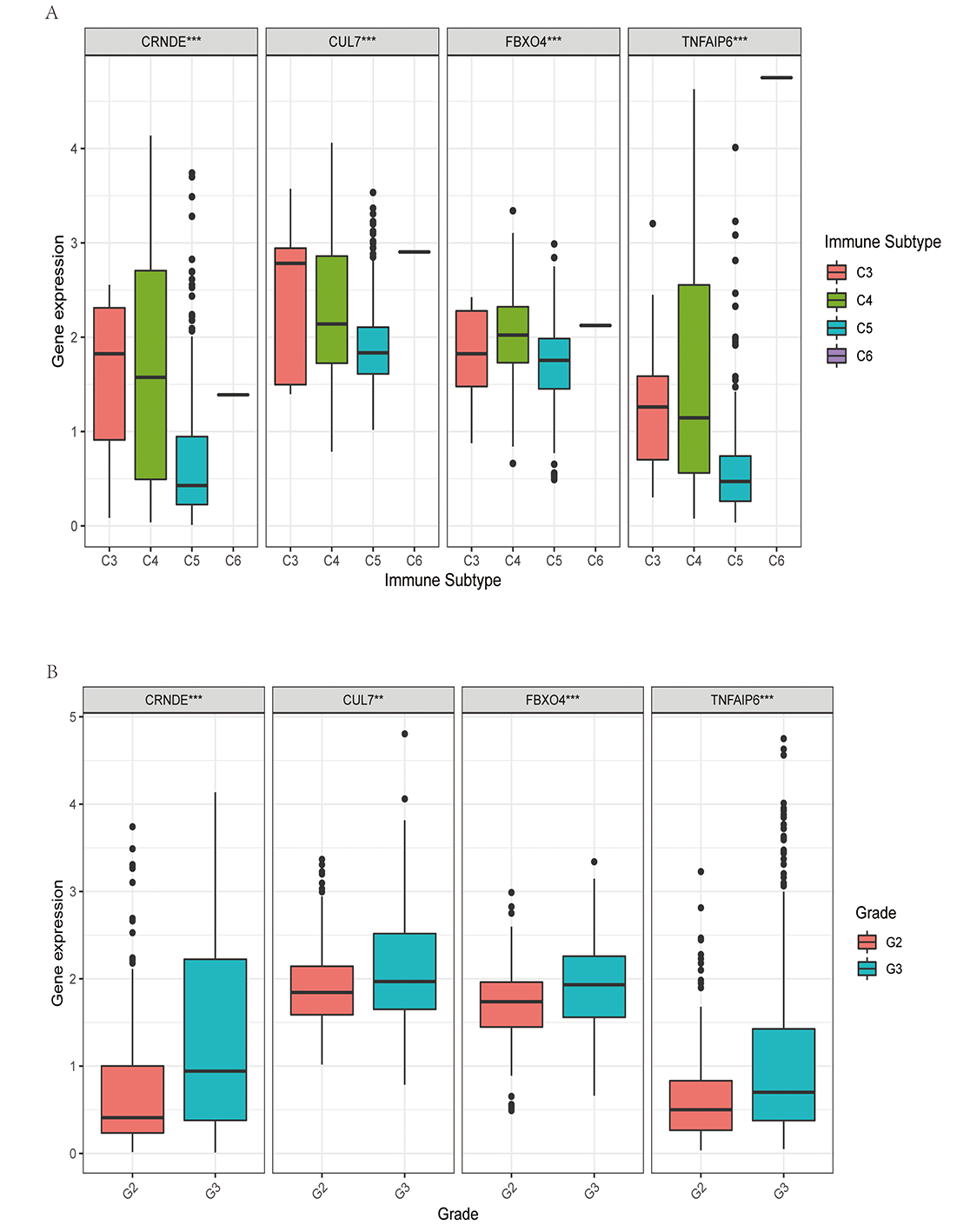

Supplement: Supplemental Information 3 — (A) Immunological subtypes; (B) clinical staging; C3, C4, C5, and C6 represent Inflammatory, Lymphocyte, Immunologically Quiet, and TGF-beta Dominant groups, respectively. [file peerj-13-19194-s003.png]

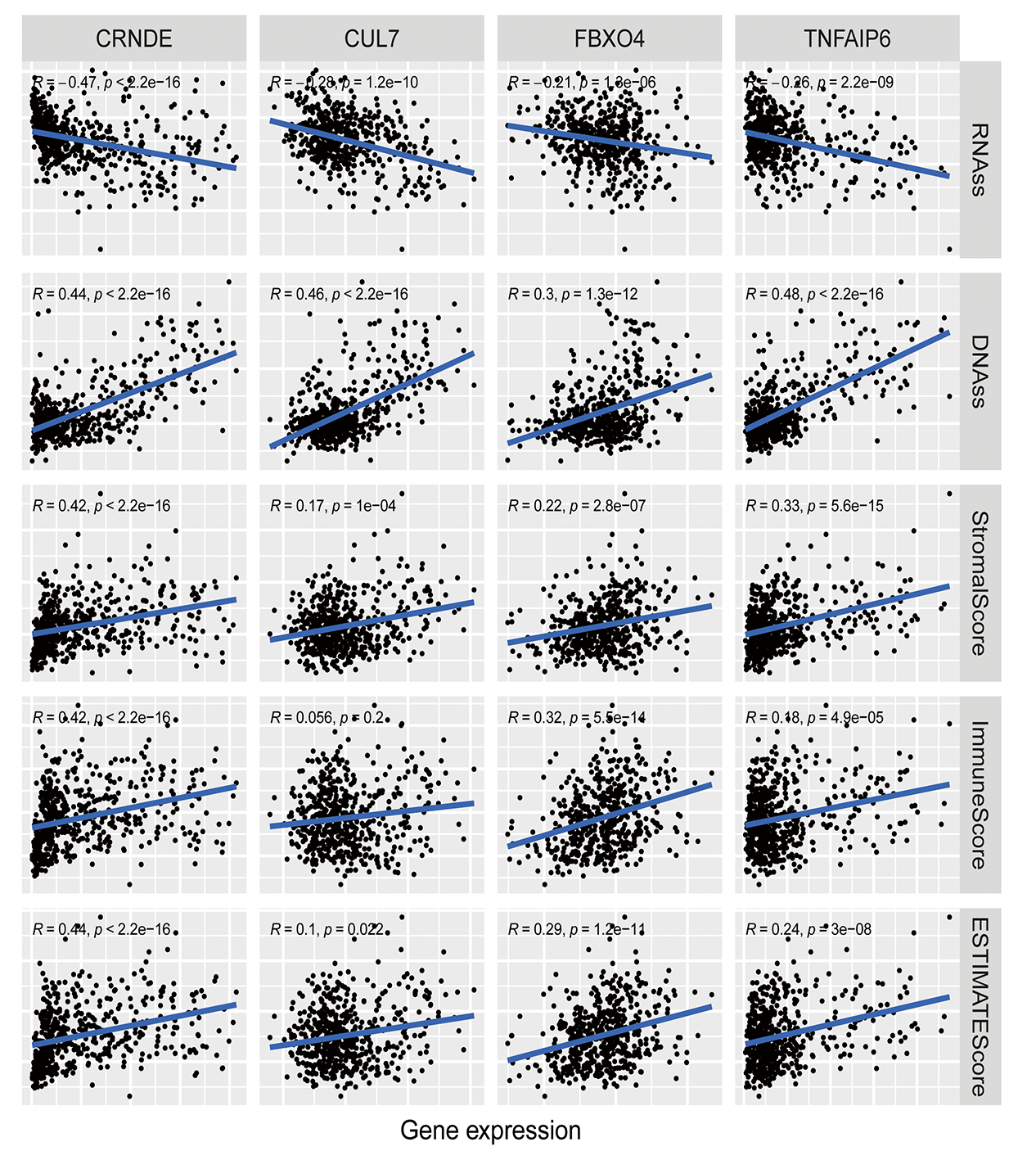

Supplement: Supplemental Information 4 [file peerj-13-19194-s004.png]

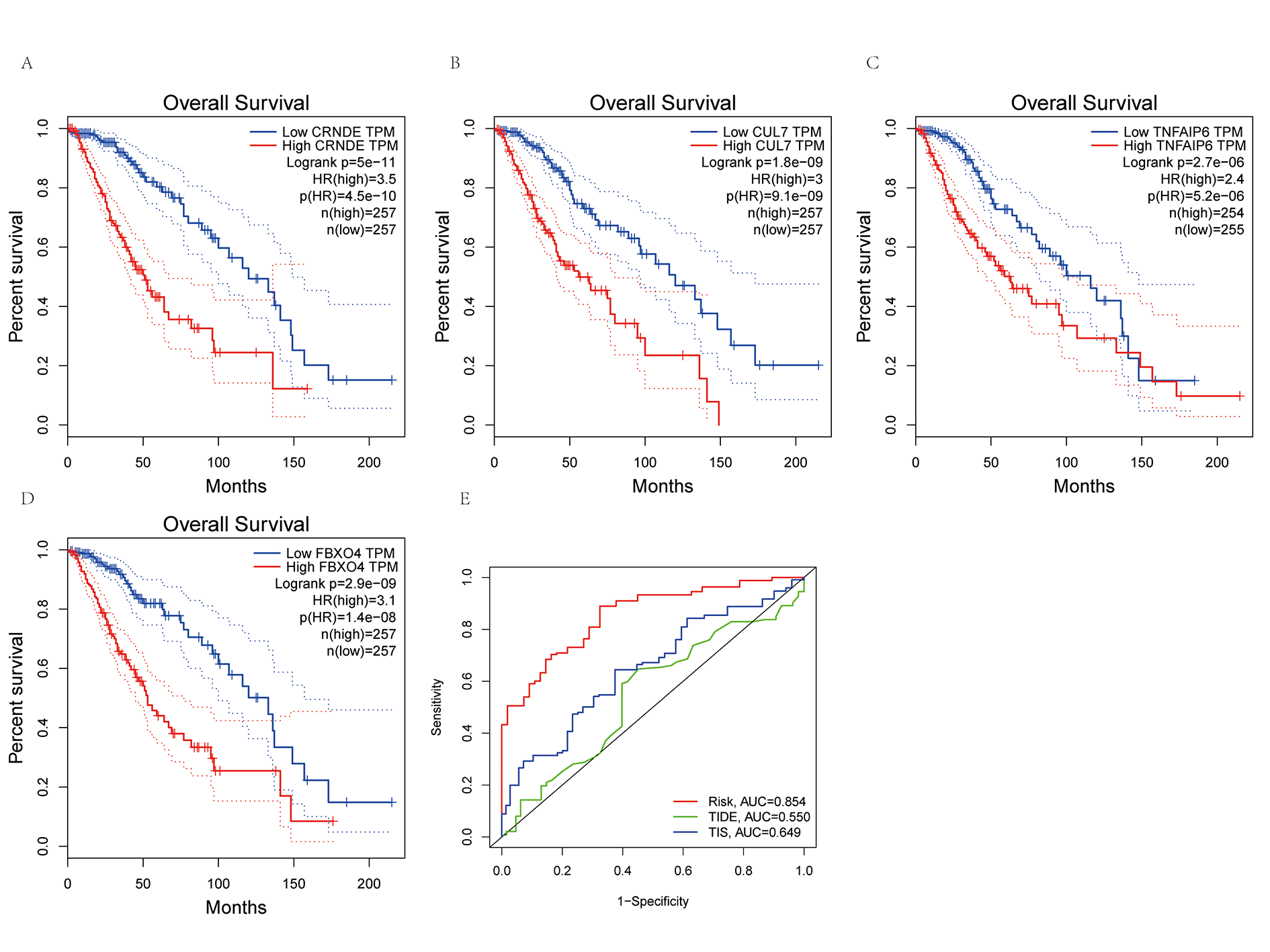

Supplement: Supplemental Information 5 — (A–D) Single-gene survival analysis, from the GEPIA website. (E) 5-year ROC curves. [file peerj-13-19194-s005.png]

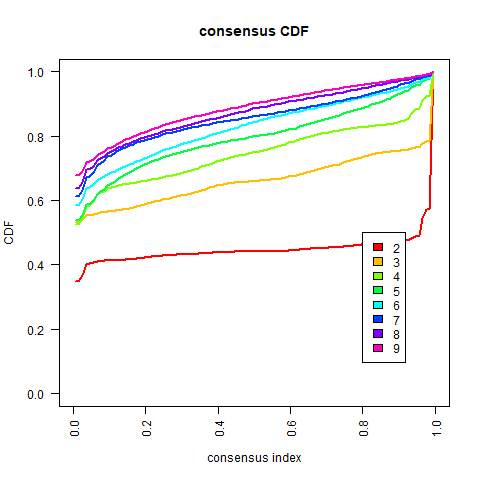

Supplement: Supplemental Information 6 [file peerj-13-19194-s006.png]

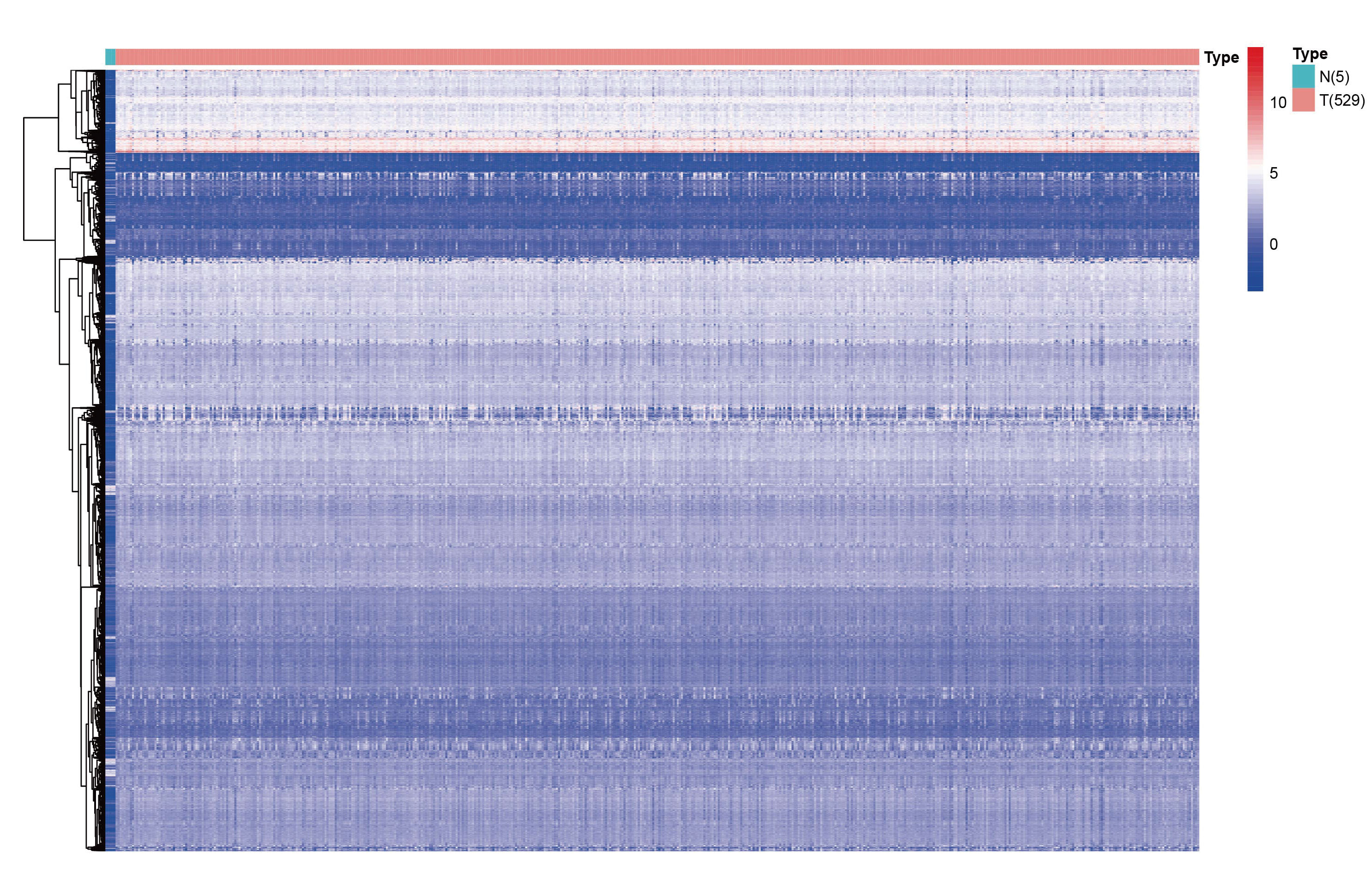

Supplement: Supplemental Information 7 — N represents normal groups (N = 5), and T represents tumor groups (N = 529). Blue represents low expression, white represents intermediate expression, and red represents. [file peerj-13-19194-s007.png]
